# Elastic Banding Compression as a Novel Treatment to Maintain Hemodynamics in a Patient With Klippel-Trenaunay Syndrome

**DOI:** 10.7759/cureus.54156

**Published:** 2024-02-13

**Authors:** Satoshi Kamiya, Takahiro Kato, Masakazu Yasuuji, Hiroyuki Tanaka, Yasuo M Tsutsumi

**Affiliations:** 1 Department of Anesthesiology and Critical Care, Hiroshima University, Hiroshima, JPN; 2 Department of Anesthesiology and Critical Care, Hiroshima City North Medical Center Asa Citizens Hospital, Hiroshima, JPN

**Keywords:** elastic bandage, pulmonary hypertension, pulmonary embolism, venous thrombosis, klippel-trenaunay syndrome

## Abstract

Klippel-Trenaunay syndrome (KTS) is also associated with venous thrombosis originating from varicose veins in the lower extremities, pulmonary embolism, and pulmonary hypertension. This study describes the anesthetic management of laparoscopic cholecystectomy in a 54-year-old male KTS patient with orthostatic hypotension due to massive varicose veins in the lower extremities and pulmonary thromboembolism. Compressing the varicosities with an elastic bandage can maintain stable circulatory dynamics even under general anesthesia management to prevent position and insufflation-induced changes that can occur spontaneously.

## Introduction

Klippel-Trenaunay syndrome (KTS) was described in 1900 as a rare congenital malformation characterized by unilateral hypertrophy of the extremities, cutaneous capillary malformation, and secondary varicosity. KTS is also associated with venous thrombosis originating from varicose veins in the lower extremities, pulmonary embolism and pulmonary hypertension secondary to thrombosis, and orthostatic hypotension due to large varicose veins [1-4]. Therefore, anesthesia management of patients with KTS requires a thorough understanding of their unique pathophysiology and close attention to respiratory and circulatory status changes. Despite this, very few reports on the anesthetic management of patients with KTS [5]. In this report, we describe the anesthetic management of laparoscopic cholecystectomy in a KTS patient with orthostatic hypotension due to massive varicose veins in the lower extremities and pulmonary thromboembolism (PTE) and chronic thromboembolic pulmonary hypertension due to thrombus formation. Ventilation, pneumoperitoneum and reverse Trendelenburg position (rTP) decrease venous return and increase pulmonary vascular resistance. We report on preoperative evaluation and intraoperative anesthesia management, including a literature review. Prior written consent was obtained from the patient.

## Case presentation

A 54-year-old male, height 169.4 cm, weight 84.4 kg, had been diagnosed with KTS due to vascular malformation of the right lower extremity since childhood. Home oxygen therapy (2L/min) was introduced due to recurrent pulmonary embolism and hypertension caused by thrombus formation. The patient developed recurrent cholecystitis due to cholelithiasis and was admitted to the hospital eight days before surgery.

An electrocardiogram showed heart rate (HR): 87 bpm sinus rhythm with no ST-segment changes or right ventricular load. Blood tests revealed total bilirubin: 1.9 mg/dL, C-reactive protein (CRP): 15.8 mg/dL, international normalized ratio of prothrombin time: 2.07, activated partial thromboplastin time: 41.9 seconds, brain natriuretic peptide: 6.0 pg/mL, D-dimer: 0.7µg/mL (Table 1).

**Table 1:** Laboratory data CRP; C-reactive protein, PT-INR; international normalized ratio of prothrombin time, APTT; activated partial thromboplastin time, BNP; brain natriuretic peptide

Variables	Patient	Reference range (adult)
Total bilirubin (mg/dL)	1.9	0.4 - 1.5
CRP (mg/dL)	15.8	0.0 - 0.3
PT-INR	2.07	0.85 - 1.15
APTT (seconds)	41.9	24 - 34
BNP (pg/mL)	6.0	0.0 - 18.4
D-dimer (µg/mL)	0.7	0.0 - 1.0

**Figure 1:**
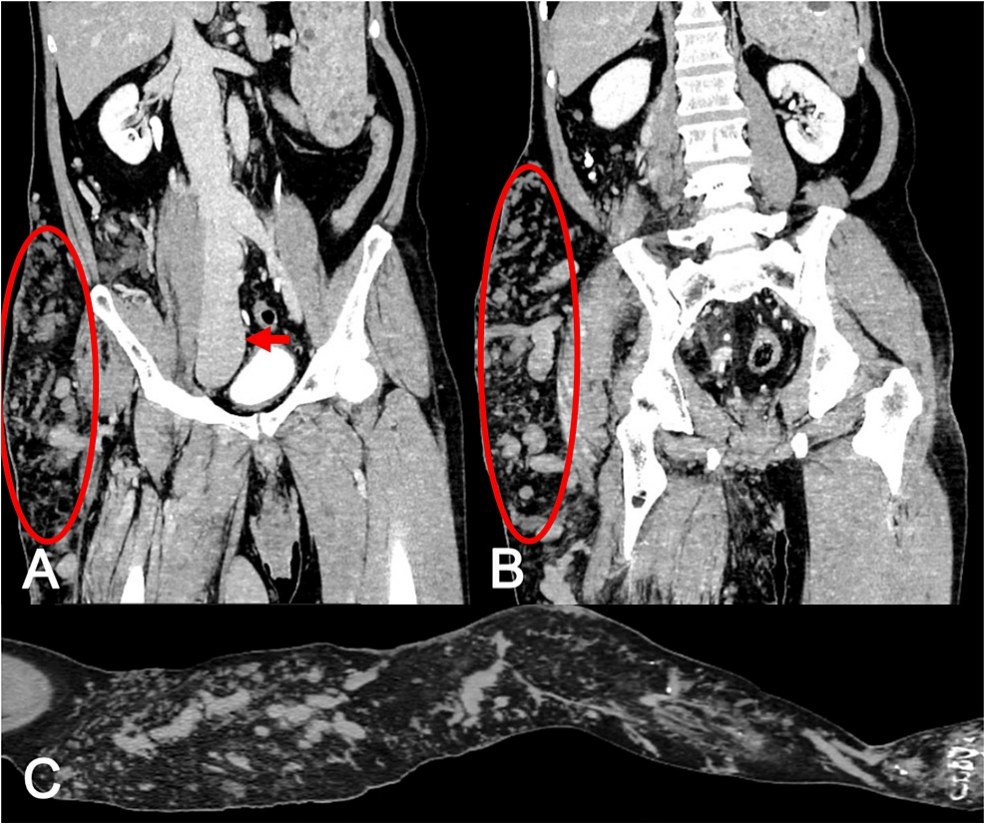
Contrast-enhanced CT. (A, B: Axial view; C: Sagittal view). Abnormally developed varicose veins can be observed mainly in the right thigh (oval). A dilated inferior vena cava can also be observed in panel A (arrow). Panel C shows a sagittal image of the right lower extremity at the level of the lateral border of the bone. In panel C, the right lower extremity at the level of the lateral border of the bone shows well-developed varicose veins, mainly in the thigh, the popliteal fossa, and the lower leg.

Chest x-ray showed a cardiothoracic ratio of 44.4% and no abnormal shadows in the lung fields. Transthoracic echocardiography revealed left ventricular ejection fraction: 62.2%, enlarged both atria, moderate ventricular hypertrophy, and trivial mitral, tricuspid and pulmonary valves regurgitation. Tricuspid annular plane systolic excursion was 21.5 mm, and the tricuspid annular peak systolic velocity was 10.6 cm/sec. Exclusion of the left ventricle at the systolic phase was observed. Contrast-enhanced computerized tomography scan showed scattered organizing thrombi observed in the peripheral areas of both pulmonary arteries. The right lower extremity showed well-developed varicose veins, mainly in the thighs. Older thrombi partially occluded several veins in the lower extremities, but no fresh thrombi formation was observed (Figures 1A-1C).

**Figure 2:**
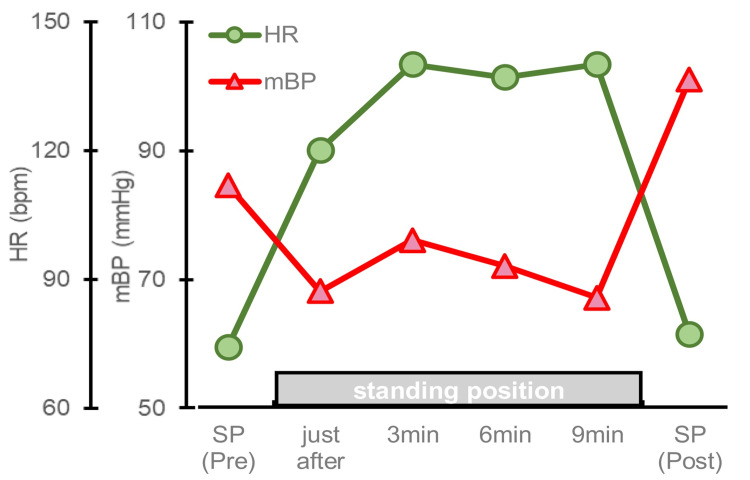
Active standing test After 10 minutes of rest in the SP, the average of the values two minutes and one minute before the start of the test was used as the pre-value. Measurements were taken immediately after active standing and at 3, 6, and 9 min. The post-value was taken 3 min after returning to the SP. Pulse rate increased with standing. Tachycardia exceeding 100 bpm continued during the test but returned to pre-test values by returning to the SP. Blood pressure decreased with standing and remained around 70 mmHg during the test. By returning to the SP, the blood pressure increased to a value higher than the pre-test value. HR – heart rate; SP – spine position; mBP – mean blood pressure.

The active standing test showed tachycardia and hypotension in the standing position (Figure 2).

Warfarin was stopped two days before surgery, and anticoagulation with heparin was performed. Before induction of anesthesia, an arterial line was inserted into the left radial artery, a pulmonary artery catheter (744HF75, Edwards Lifesciences, Irvine, America) was inserted through the right internal jugular vein, and Vigilance II® (VIG2, Edwards Lifesciences, Irvine). Continuous cardiac output (CCO), continuous cardiac index (CCI), and mixed venous blood oxygen saturation (SvO2) measurements were initiated with propofol TCI: 3.0µg/mL, remifentanil: 0.4 µg/kg/min, and rocuronium: 50 mg. Tracheal intubation was carried out with cuffed endotracheal tube with an inner diameter of 8.0 mm using a video laryngoscope (McGRATH MAC, Medtronic Inc, Minneapolis, MN, USA).

**Figure 3:**
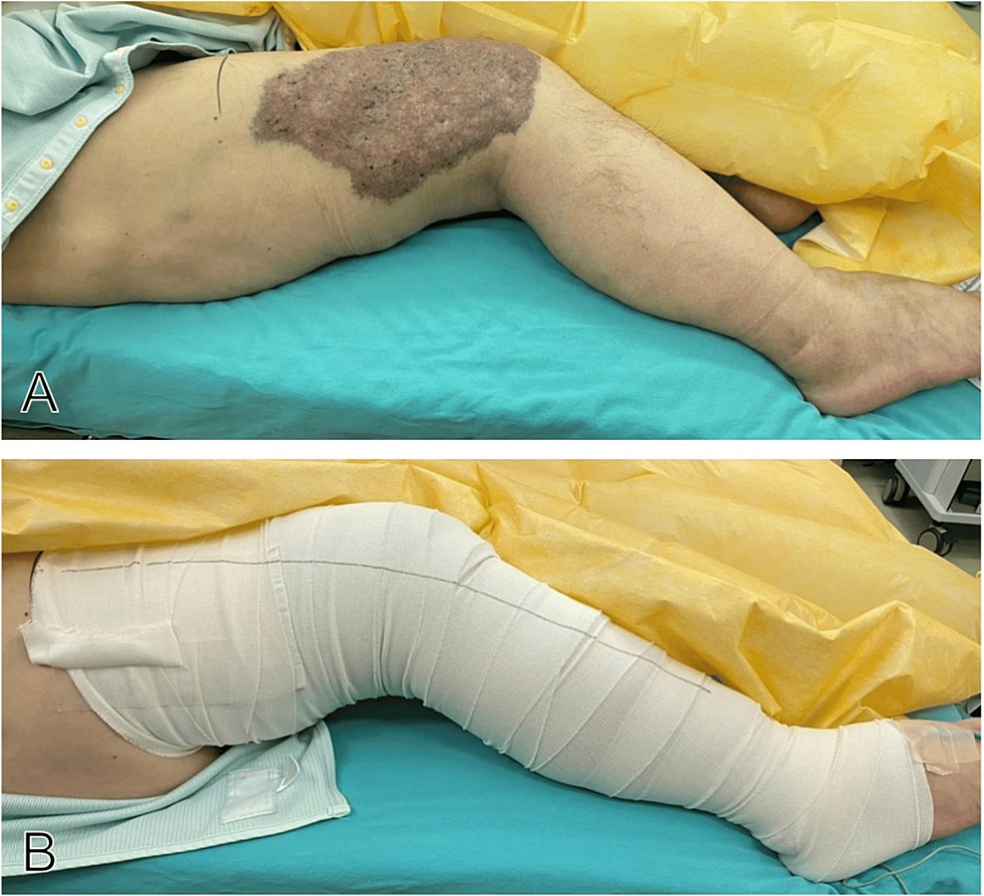
Elastic band compression (EBC) application A: Before EBC, B: After EBC. The femoral circumference from the superior border of the patella to the four lateral phalanges was 53.0 cm on the right (affected side) and 39.5 cm on the left (healthy side) before EBC. In B, the LDF sensor can be seen affixed to the dorsum of the foot. LDF – Laser Doppler flowmetry.

Compression of the affected limb was performed with an elastic bandage before the induction of general anesthesia. The bandage was rolled with the same pressure as general elastic compression stockings, allowing one finger to be inserted between the patient's body surface and the bandage if it is strongly stretched. To prevent low blood flow due to excessive high pressure, laser Doppler flowmetry (LDF) was attached to the dorsum of the foot for continuous monitoring (Figures 3A, 3B). 

**Figure 4:**
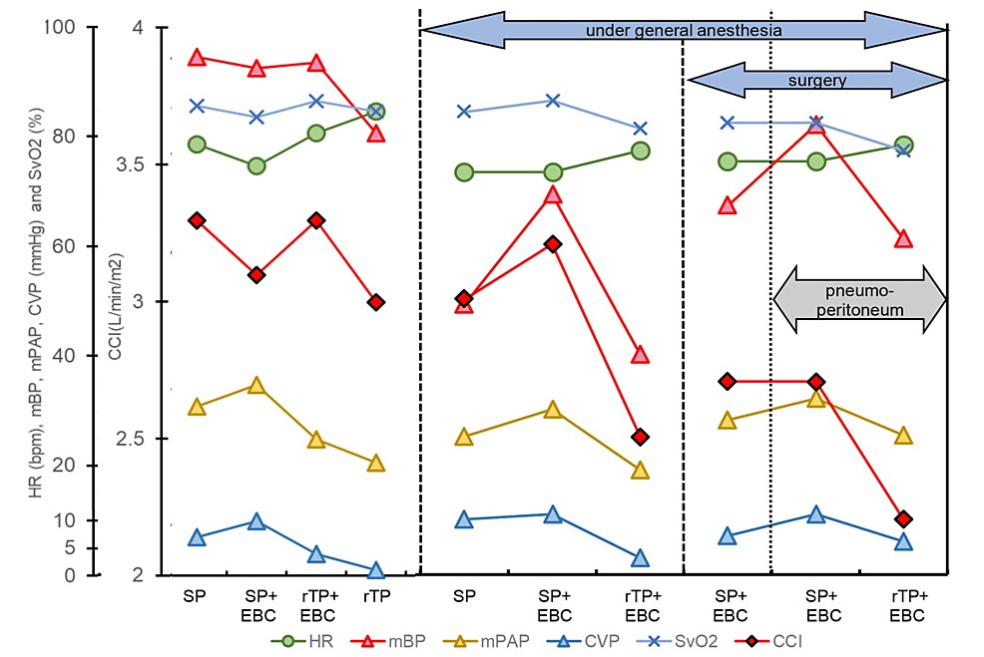
Hemodynamic measurements The first panel is recorded in the supine position before induction of anesthesia; elastic band compression (EBC) increased central venous pressure (CVP) but decreased both mean blood pressure (mBP) and continuous cardiac index (CCI). When reverse Trendelenburg position (rTP) was performed, CVP decreased, but mBP and CCI increased. rTP and removal of EBC resulted in a 14.9% decrease in mBP, from 94 to 80 (mmHg). During the maintenance phase of anesthesia, both mBP and CCI increased along with the increase in CVP due to EBC. Repositioning from supine to rTP, both CVP, mBP, and CCI decreased. SP – supine position; HR – heart rate, mPAP – mean pulmonary artery pressure, SvO_2_ – venous oxygen saturation.

The changes in circulatory index with and without elastic bandage compression (EBC) of the affected limb were observed in spine position (SP) and 10° rTP before and after induction of anesthesia, respectively. Anesthesia was maintained with propofol TCI: 2.0 µg/mL + remifentanil: 0.3 µg/kg/min. Phenylephrine was administered to treat hypotension during maintenance of anesthesia with a bolus dose of 0.1mg twice and a continuous dose of 1mg/h. The circulatory index was recorded with EBC even after 10mmHg insufflation. A list of changes in the circulatory index at each point where recordings were made (Figure 4).

After the abdominoscopy and changed to SP, the EBC was released. The patient was extubated and admitted to the ICU after confirming that his circulation was stable. The total operation time was 2 hours and 44 minutes, and the anesthesia time was 4 hours and 33 minutes. Intraoperative fluid balance was +410 mL (crystalloid: 530 mL; urine output: 60 mL, blood loss: 60 mL).

On the first post-operative day, the patient resumed eating, restarted warfarin, and started continuous intravenous infusion of heparin. In addition, orthostatic changes (blood pressure (BP): 105/50 → 70/30 mmHg) and presyncope were observed. On the third postoperative day, he was able to hold a standing position without feeling dizzy and ambulate with a walker. On the fifth postoperative day, a contrast-enhanced CT scan showed small new thrombi in the upper lobe branch of the right pulmonary artery and a vein in the right soleus muscle. Still, the patient continued to receive continuous heparin, and his respiratory status and hemodynamics were maintained without deterioration.

## Discussion

Anesthesia for laparoscopic cholecystectomy in a patient with KTS requires careful maintenance of venous return, as large intraoperative circulatory fluctuations were expected due to chronic thromboembolic pulmonary hypertension and orthostatic hypotension. The patient's BP decreased during the induction of anesthesia and rTP but increased by compression with an elastic bandage. Stable circulatory control was maintained during surgery.

Efficacy of compression to maintain hemodynamics

In the anesthetic management of patients with pulmonary hypertension, it is essential to maintain adequate systemic vascular resistance (SVR) to prevent right ventricular ischemia and to maintain adequate venous return to avoid right heart failure due to elevated pulmonary artery pressure (PAP) [6]. As with induction of anesthesia, rTP and pneumoperitoneum decrease venous return [7]. However, if the temporary decrease in venous return during laparoscopy is adapted only by infusion, there is a risk of relative excess venous return after insufflation and rTP is completed. In addition, most cardiovascular agonists increase pulmonary vascular resistance, so care should be taken to avoid elevated pulmonary arterial pressure.

On the other hand, EBC, which has long been used as a treatment for varicose veins, has been reported to decrease venous volume and lose its effect after pressure release [8]. This may be useful for maintaining circulatory dynamics in response to a temporary decrease in venous return.

Safety of EBC on the affected extremity

In this case, anesthesia management was performed with compression of the affected limb, a high-risk site for thrombus formation, and the patient had a stable course without any circulatory or respiratory status changes that would raise suspicion of acute pulmonary embolism after the release of compression. Several precautions must be taken to ensure the patient's safety during limb compression. Elastic stockings are recommended in the perioperative period to prevent deep vein thrombosis (DVT). However, the pros and cons of wearing elastic socks for acute DVT are inconclusive [9]. Some reports suggest that 9% and 2% of KTS patients have a history of DVT and PTE, respectively [5]. However, the incidence of perioperative DVT and PTE remains unknown. Prevention of DVT and PTE is critical, as KTS patients may be more prone to DVT than healthy individuals. In this case, we could confirm no acute DVT or PTE by performing a contrast-enhanced CT scan two days before the procedure. We believe this preoperative confirmation allowed us to compress the lower extremity safely.

Concern for impaired blood flow secondary to compression

Elastic stockings may improve venous blood flow while impairing arterial blood flow. In this case, LDF was used to confirm that there was no decrease in peripheral blood flow due to compression of the affected limb; changes in LDF measurements have been reported to have the same significance as changes in blood flow and clinical findings [10]. Elastic bandage has the advantage that the compression pressure can be easily changed by rewrapping, but it is difficult to measure the compression pressure and the possibility of excessive pressure should be noted by continuously confirming that peripheral blood flow in the affected limb has not decreased.

Several limitations exist in this report. First, nitric oxide, which selectively dilates pulmonary vessels, could not be used due to the limitations of the hospital facilities. The reduction in right heart afterload may have prevented the decrease in CCI due to ventricular interdependence and helped maintain more stable circulatory dynamics. Second, this is a single case report. Although the increase in venous return due to EBC is expected to be greater in KTS than in normal subjects, the results may not be the same in other similar patients because of individual differences in the degree of varicose veins and the volume of circulating blood. Further validation is needed to generalize this to other patients.

## Conclusions

We experienced a laparoscopic cholecystectomy for a patient with KTS with chronic thromboembolic pulmonary hypertension and orthostatic hypotension. Compressing the varicosities with an elastic bandage maintained stable circulatory dynamics even under general anesthesia. In KTS patients with impaired physiologic compensatory function of the right ventricular system, we suggest that the assumption of changes in circulation dynamics and EBC adjustment are effective for safe anesthesia management to prevent position and insufflation-induced changes that can occur rapidly.
